# A 1-GHz 64-Channel Cross-Correlation System for Real-Time Interferometric Aperture Synthesis Imaging

**DOI:** 10.3390/s19071739

**Published:** 2019-04-11

**Authors:** Xiangzhou Guo, Muhammad Asif, Anyong Hu, Zhiping Li, Jungang Miao

**Affiliations:** School of Electronic Information Engineering, Beihang University, No. 37, Xueyuan Road, Haidian District, Beijing 100191, China; xiangzhou.guo@outlook.com (X.G.); m.asif.bhatti@gmail.com (M.A.); zhiping_li@buaa.edu.cn (Z.L.); jmiaobremen@buaa.edu.cn (J.M.)

**Keywords:** interferometry, aperture synthesis, security screening, cross correlator, 1-bit, comparator, FPGA

## Abstract

We present a 64-channel 1-bit/2-level cross-correlation system for a passive millimeter wave imager used for indoor human body security screening. Sixty-four commercial comparators are used to perform 1-bit analog-to-digital conversion, and a Field Programmable Gate Array (FPGA) is used to perform the cross-correlation processing. This system can handle 2016 cross-correlations at the sample frequency of 1GHz, and its power consumption is 48.75 W. The data readout interface makes it possible to read earlier data while simultaneously performing the next correlation when imaging at video rate. The longest integration time is up to 68.7 s, which can satisfy the requirements of video rate imaging and system calibration. The measured crosstalk between neighboring channels is less than 0.068%, and the stability is longer than 10 s. A correlation efficiency greater than 96% is achieved for input signal levels greater than −25 dBm.

## 1. Introduction

Interferometric aperture synthesis has long been used in radio astronomy and earth remote sensing [1,2], utilizing a sparse array of antennas to synthesize a large aperture. Interferometers measure the complex cross correlations between signals received by each pair of antennas to get the so-called visibility function samples. According to the Van Cittert–Zernike theorem [3], the brightness temperature within the field of view (FOV) can be approximated by the inverse Fourier transform of the visibility function samples.

In the recent past, this technique has been found useful for several other applications, including passive millimeter wave (PMMW) imaging for security screening [4,5]. Several two-dimensional (2-D) interferometric aperture synthesis demonstrators have been developed [6,7,8], which verify its ability to detect threats and show the advantages of its high imaging rate and large FOV. However, PMMW imaging systems suffer from low contrast between the targets and background in an indoor environment, which need a radiometric sensitivity of less than 1 Kelvin to enable the detection of metallic and non-metallic threats on human bodies. A 2-D interferometric aperture synthesis PMMW imager with adequate radiometric sensitivity and spatial resolution at the video imaging rate needs several hundred antenna-receiver channels with a bandwidth of more than 1 GHz [4], and both the complexity and the cost are too high to build the correlation processing subsystem at this stage.

**Figure 1:**
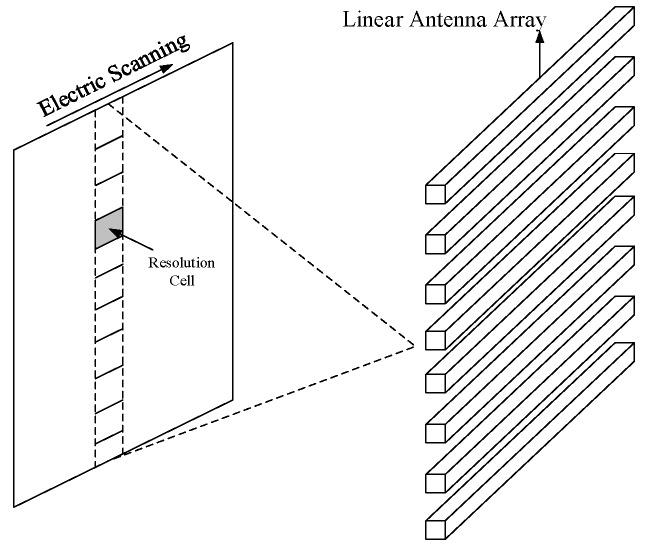
The hybrid architecture of phased array and aperture synthesis.

**Figure 2:**
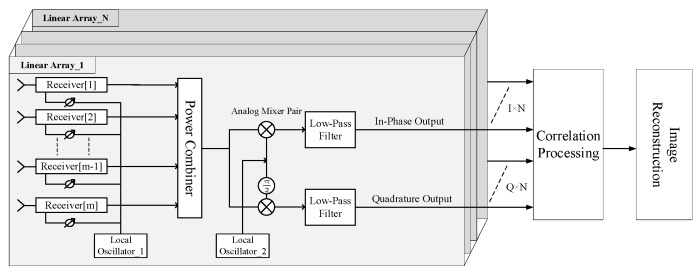
Signal processing flow of the hybrid system.

A hybrid passive imaging architecture is proposed in Beihang University to reconcile the requirements for radiometric sensitivity, spatial resolution, and imaging rate with the complexity of signal processing. As shown in Figure 1, the hybrid architecture uses linear phased arrays to obtain resolution in one direction and use aperture synthesis to obtain resolution in the cross direction. Each linear antenna array produces a fan beam by the phased array approach which is narrow in the direction of electric scanning and broad in the cross direction. The fan beam scans along one direction in the FOV and provides resolution along this direction. At each pointing of the fan beam, resolution across the scanning direction is achieved using aperture synthesis. Figure 2 illustrates the signal processing employed in the hybrid system. The signal collected by each antenna is filtered, amplified, and mixed to a convenient intermediate frequency by a heterodyne receiver. The local oscillator signal for each receiver is configured with a different phase according to the pointing of the fan beam. Then, the signals from the antenna–receiver channels in the same linear array are connected to a power combiner to form a fan beam. The output signal of each power combiner is connected to an analog mixer pair to generate the complex analytic signal consisting of in-phase and quadrature (IQ) outputs at the baseband. Correlation processing, including cross-correlation and self-correlation, is performed on these complex signals to get visibility function samples. Finally, the visibility function samples are used to reconstruct an image.

**Figure 3:**
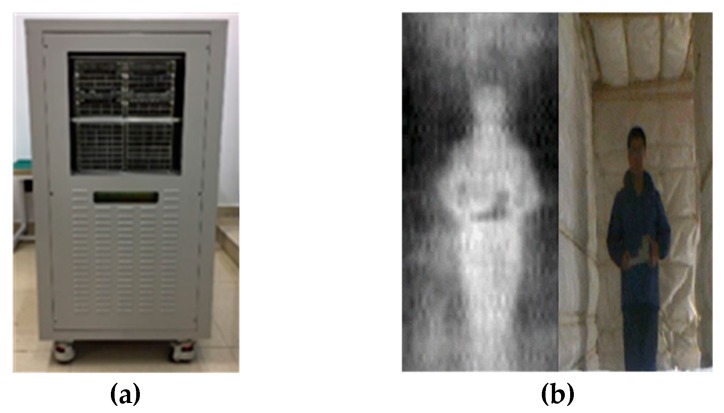
(**a**) A 256-channel demonstrator employing the hybrid architecture; (**b**) An image of a person holding a metallic gun model under an artificial hot background with a temperature of about 573 Kelvin.

The hybrid architecture is similar to that of ESTAR [1], for which aperture synthesis only has to be done in one dimension. However, due to electric scanning, the integration time for aperture synthesis in the hybrid system is much shorter than that in a 2-D aperture synthesis system, and a large bandwidth is needed to satisfy the radiometric sensitivity requirement. A Ka-band 256-channel demonstrator with 1 GHz bandwidth employing the hybrid architecture has been built, as shown in Figure 3a. This uses a 16 × 16 metal horn antenna array and 64 analog complex correlators [9]. Figure 3b is an image of a person holding a metallic gun model that was obtained under an artificial hot background with a temperature of about 573 Kelvin, where the measured radiometric sensitivity was 2 Kelvin. The hot background is specially designed to improve the contrast between the human body and a non-metallic object that has similar emissivity to human tissues. If the emissivity of an object only has a difference of 0.01 to that of human tissues, an ambient temperature of 573 K will lead to an equivalent radiometric temperature difference of about 2.7 K, which can be detected by the imager.

An enhanced system is now under development, which aims to improve radiometric sensitivity by increasing the bandwidth to 4 GHz and achieve a better spatial resolution by moving to the Q band. To suppress the degradation of spatial resolution at oblique viewing angles caused by fringe washing, the band division correlation (BDC) technique [10] is used to build the correlation processing subsystem. The received signals with 4 GHz bandwidth are divided evenly into four sub-bands of 1 GHz to reduce decorrelation effects. The enhanced imager uses a 32 × 32 antenna array, for which 1984 complex correlators are needed. Although some efforts have been made to realize the analog correlator in a compact size [9], the total size is still too large for such a number of analog correlators. Thus, digital correlators are adopted in this system.

The digital correlators can be implemented using FPGA [11], graphics processing units (GPUs) [12], and application-specific integrated circuits (ASICs) [13,14,15,16]. GPUs use a peripheral component interconnect express (PCIe) interface to communicate with peripherals, which cannot connect with analog-to-digital convertors (ADCs), and lead to extra complexity when capturing sampled results. Although ASICs can offer an excellent performance and extremely low power dissipation, the cost is too high, and there is no strict constraint on power dissipation for ground-based applications. State of the art FPGAs have flexible interfaces to communicate with peripherals and abundant logic resources to perform signal processing, which is suitable for our system.

This paper presents a 64-channel digital correlation system that performs 1-bit/2-level (1B/2L) digitization and correlation processing at 1 GHz. The 1B/2L digitization is achieved using commercial comparators and the correlation processing is achieved on FPGA. Because 1B/2L digital correlators can only measure the correlation coefficients of input signals, this system also integrates power measurement circuits for each channel, and visibility samples are derived by anti-normalizing correlation coefficients using power measurement results. Based on this 1 GHz system, we can build a system with 4 GHz bandwidth using BDC technology.

## 2. Cross-Correlation Theory

**Figure 4:**
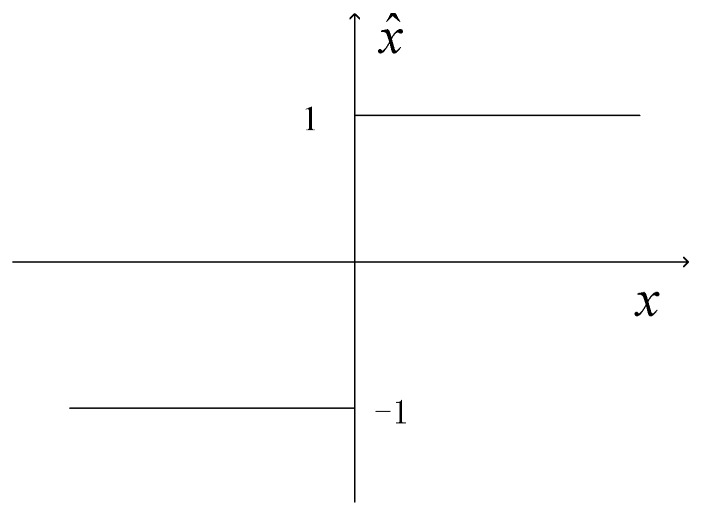
Characteristic curve for two-level quantization. The abscissa is the input voltage x and the ordinate is the quantized output $\hat{x}$.

The signals measured by the passive imager were spontaneous electromagnetic radiations of the object. The input signals of the correlator for a given target could be formulated as two stationary, ergodic, zero mean Gaussian random processes, and the joint probability density function (PDF) was formulated by

(1) $$p(x,y)=\frac{1}{2\pi \sigma^{2}\sqrt{1-\mu^{2}}}\exp\left\{-\frac{x^{2}+y^{2}-2xy\mu }{2\sigma^{2}(1-\mu^{2})}\right\},$$

where σ is the standard deviation of the signals, and μ is the correlation coefficient. A digital correlator consists of a multiplier followed by an accumulator that sums the products of the input samples. The quantization characteristic for 1B/2L sampling is shown in Figure 4, where input signals are assigned values of +1 or −1 to indicate positive or negative signal voltages. If sampling is at the Nyquist rate and the number of sample pairs fed to the correlator is N, the two-level correlation coefficient is

(2) $$\mu_{2}=\frac{\left(N_{11}-N_{\bar{1}\bar{1}})-(N_{\bar{1}1}+N_{1\bar{1}}\right)}{N},$$

where $N_{11}$ is the number of products for which both samples have the value +1, $N_{1\bar{1}}$ is the number of products in which the x sample has the value +1 and the y sample −1, and so on. $\mu_{2}$ can be related to the correlation coefficient μ of the unquantized signals through the bivariate probability distribution as Equation (2) [17], which can be expressed by

(3) $$\mu_{2}=\frac{2}{\pi }\sin^{-1}\mu .$$

Equation (4), known as the Van Vleck relationship [18], allows μ to be obtained from the measured correlation $\mu_{2}$. For small values, μ is proportional to $\mu_{2}$. In order to calculate the visibility function samples, the correlation coefficient needs to be denormalized by

(4) $$V=T_{sys}\mu ,$$

where $T_{sys}$ is the system noise temperature. $T_{sys}$ could be obtained by measuring the output power of the receiver.

As 1B/2L quantization senses only the sign of the instantaneous signal voltage and loses the power information, it has the worst quantization efficiency, 0.64 [17]. On the other hand, 1B/2L can be achieved using comparators, which makes it easy to obtain a high compactness and a low power consumption. The theoretical radiometric sensitivity can be estimated using the standard radiometer equation adapted for aperture synthesis, which is given by

(5) $$\Delta T=\frac{T_{A}+T_{R}}{\sqrt{B_{RF}\times t_{INT}}}\frac{1}{F\eta_{Q}\eta_{M}},$$

where $T_{A}$ is the antenna temperature (~300 K in an indoor environment), $T_{R}$ is the noise temperature of the whole receiver chain (~300 K for well-designed receivers), $B_{RF}$ is the radio frequency (RF) bandwidth, $t_{INT}$ is the integration time, $\eta_{Q}$ is the sampling quantization efficiency, $\eta_{M}$ is the antenna main beam-efficiency (typically 80% for a well-designed antenna) and F is the fractional filling of the array (80% for the 32 × 32 array). The enhanced system is designed to work at an imaging rate of 15 frames per second and each frame includes 80 beam paintings, where the integration time for each beam pointing is 0.83 ms. When 1B/2L quantization is adopted, a radiometric sensitivity of about 0.8 Kelvin can be obtained, with 4 GHz bandwidth.

## 3. Architecture

**Figure 5:**
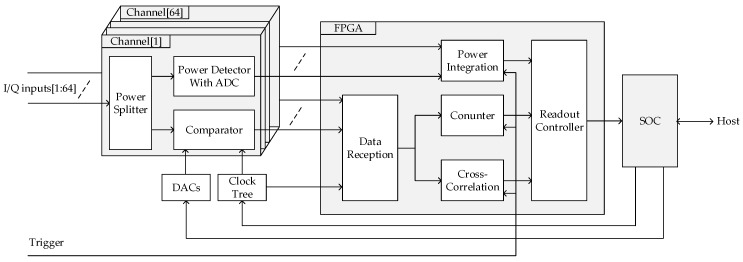
Block diagram of the correlation system.

The block diagram of the 64-channel digital correlation system is shown in Figure 5. Every analog input signal was first connected to a two-way power splitter to generate two copies: one was sampled by a comparator at 1 GHz, the other was fed to a power detector for which the output was sampled with a 16-bit ADC. The 1-bit sampled data and the digital power detection results were captured by an FPGA to perform further processing. The threshold offset per comparator was calibrated using a digital-to-analog convertor (DAC). For common comparators, which have no source-synchronized clock for data reception, a data reception interface was designed, including a clock tree and a data reception module in the FPGA. Because of the hybrid imaging architecture, an external trigger signal was routed to the FPGA to synchronize the correlation operation with the phased array scanning. A system on chip (SOC) device was used to control all the other devices in this module and communicate with the host.

### 3.1. High-Speed One-Bit Digitization with Adjustable Threshold

**Figure 6:**
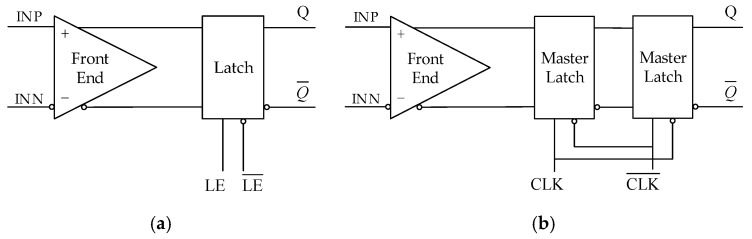
Comparator circuit topologies: (**a**) level-latched comparator; (**b**) edge-latched comparator (or clocked).

1B/2L digitization can be achieved using comparators. As shown in Figure 6, the comparators used for 1-bit quantization can be divided into two categories: the level-latched comparators and the clocked or edge-latched comparators. The reason for using edge-latched comparators instead of level-latched comparators is that edge-latched comparators hold data outputs stable for an entire clock period, making it easier for the FPGA to capture the sampled data.

When a 1B/2L correlator is adopted, errors during digitization are mainly from two sources: the threshold offset of the comparator and timing skew between sampling clocks. The threshold offset of comparators are compensated using DACs with 0.1 mv resolution, which makes it possible to obtain a normalized threshold offset of about 0.4% with −25 dBm noise input. As shown in Figure 5, a counter module was implemented in the FPGA, which counted the total number of one-bit samples and the number of “ones” in each data channel. When a Gaussian white noise was fed to the input port, the SOC could adjust the threshold according to percentage of “ones” in all samples. The procedure to adjust the threshold worked as follows. If the percentage of “ones” was larger than 0.5, then the actual threshold was lower than zero and the DAC output would increase by one step. If the percentage of “ones” was smaller than 0.5, then the actual threshold was higher than zero and the DAC output would decrease by one step. Repeating this operation, the threshold would converge to zero.

**Figure 7:**
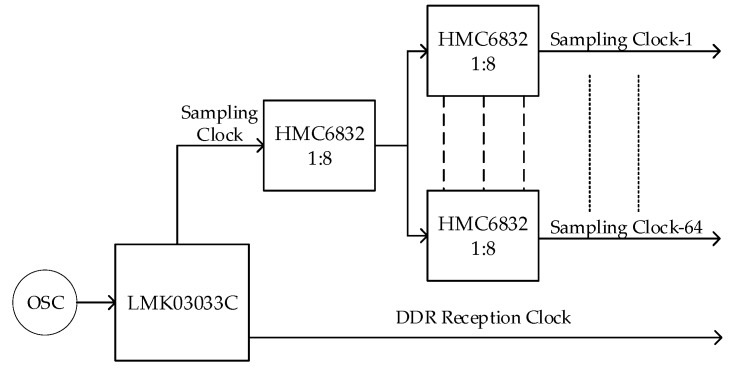
Schematic of the clock tree.

Timing skew between sampling clocks has an effect of reducing the correlator output, which can be expressed by [19]

(6) $$\Delta \gamma =1-\frac{\sin(2\pi B\Delta t)}{2\pi B\Delta t}$$

where Δγ is the percentage of reduction in correlator output caused by the timing skew, B is the bandwidth of input white noise signal, and Δt is the skew between sampling clocks. To guarantee the radiometric sensitivity, the reduction should be less than 5%, which can be caused by $\Delta t=0.087B^{-1}$. For a 500 MHz bandwidth, this corresponds to Δt=174 ps. The schematic of the clock tree is shown in Figure 7. A clock conditioner LMK03033C [20] was used to generate a 1 GHz sampling clock and a double data rate reception clock of 500 MHz. As the signal-channel comparator HMC874 [21] was used, a two-stage clock distribution network was built using the low-skew clock buffer HMC6832 [22] to generate 64 1 GHz synchronous sampling clocks. The sampling skew was less than 46 ps, leading to a reduction of less than 0.5% for 500 MHz bandwidth.

### 3.2. Data Reception in FPGA

There are three issues with the FPGA capturing the sampled data. First, it is impossible for the FPGA to do cross-correlation processing synchronous with a clock up to 1 GHz, and a mechanism like serial-to-parallel conversion is needed to reduce the working frequency without data loss. Second, common comparator chips have no source-synchronous clock output for data reception as the ASIC presented in [23], while the sampled data have to be synchronized to the same clock domain to perform cross-correlation. Third, pin variations of the FPGA will introduce non-negligible bit skew when synchronizing tens of data lines across several banks to the same clock domain at 1 GHz, even though the arriving data are totally synchronous at the input/output (IO) pins of the FPGA. The bit skew may cause setup time and hold time violation for the FPGA to capture the incoming data if not calibrated, which will lead to a large amount of error bits and reduce the measurement signal-to-noise ratio.

**Figure 8:**
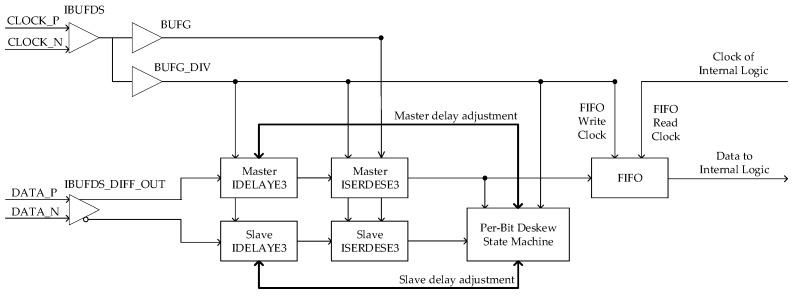
Schematic of the data reception module.

A data reception module was implemented in the FPGA, which performed per-bit deskew and 1:4 serial-to-parallel conversion. This module was designed based on the delay component IDELAYE3 and the deserialization component ISERDESE3 available in Xilinx Kintex UltraScale FPGA [24]. The IDELAYE3 can delay any input signal except global clocks, and the ISERDESE3 can avoid the additional timing complexities encountered when designing deserializers in the device logic [25]. The schematic of a data reception channel is shown in Figure 8. Because the maximum input clock frequency of the general IO interface was less than 1 GHz, a double data rate (DDR) reception method was adopted, where the frequency of the receiver clock was equal to half that of the sampling clock, as shown in Figure 7.

The DDR clock was routed from a global clock input pin-pair to both the global clock buffers, i.e., BUFG and BUFG_DIV, via the IBUFDS input buffer. The BUFG_DIV divided the input clock by n, where n is half of the required serial-to-parallel rate, that is 1:4 or 1:8. The BUFG clock was used to sample the serial data at the input of ISERDESE3, while the BUFG_DIV was used to clock parallel data out of the ISERDESE3 and clock the per-bit deskew state machine. The output of the BUFG was also used to clock the user logic. The incoming differential data lines were routed to a master IDELAYE3 and a slave IDELAYE3 via the IBUFDS_DIFF_OUT input buffer. After delay adjustment, these signals were connected to the master and slave ISERDESE3s. Parallel data from the master ISERDESE3 was forwarded into the per-bit deskew state machine and into the internal logic via first-in/first-out (FIFO) memory. Parallel data from the slave ISERDESE3 was only used by the per-bit deskew state machine.

**Figure 9:**
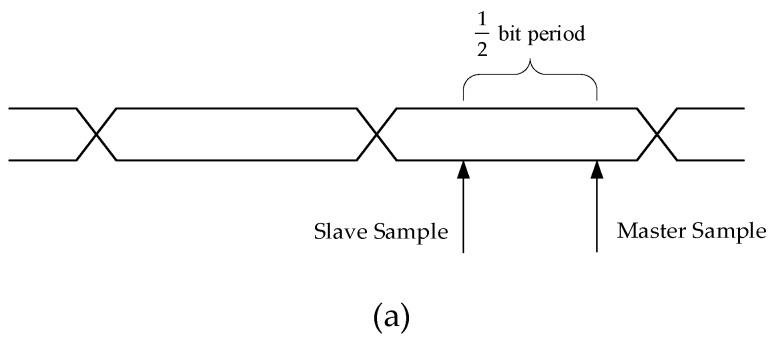

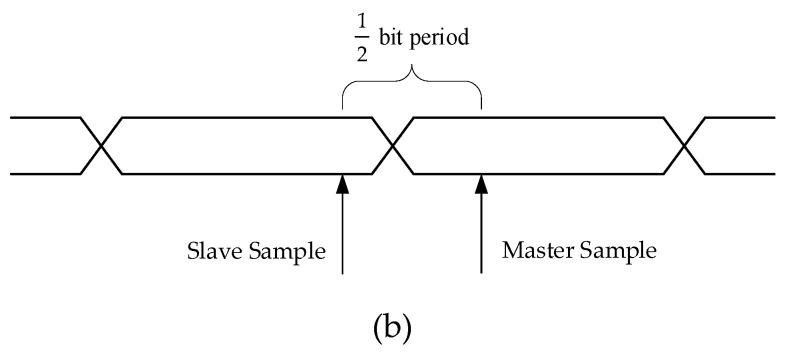
(**a**) Master sampling point is too late in a bit period. (**b**) Master sampling point too early in a bit period.

Following a power-up or reset, the per-bit deskew state machine started running. The algorithm used to perform per-bit deskew originated from [26] and has been used in source-synchronous interfaces [27], which work as follows. If the two samples taken were half a bit period apart (following a transition) and were the same, then the master sampling point was too late, as shown in Figure 9a, and the input data delay needed to be increased by one step. If the two samples taken (following a transition) were different, then the master sampling point was too early, as shown in Figure 9b, and the input data delay needed to be decreased by one step.

The initial value of the master data delay was set to only compensate for the data-to-clock skew resulting from printed circuit board (PCB) routing and chip propagation delays, which is easy to obtain from a fixed PCB design. This ensured that the initial sample point was almost positioned in the correct place, and the per-bit deskew state machine was used to fine-tune each data line from that point onwards to improve data reception performance.

### 3.3. Correlation Processing

There were 32 channels in the aperture synthesis dimension of the hybrid system, and each channel had a pair of IQ outputs with 500 MHz bandwidth. To achieve full cross-correlation of 32 complex signals in each sub-band, 496 complex cross-correlations or 1984 real cross-correlations should be measured simultaneously at 1 GS/s. Besides this, 32 additional correlations between IQ outputs inside one channel were calculated, which were used to calibrate the quadrature error. So, the correlation system must be able to handle 2080 real correlations at the sample rate of 1 GS/s.

**Figure 10:**
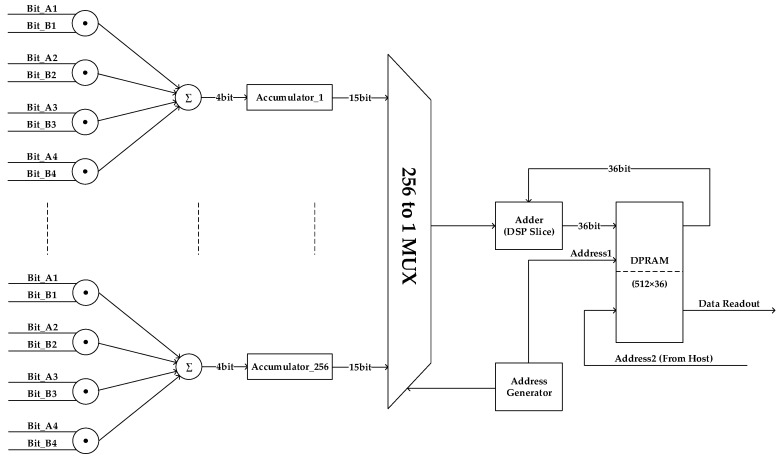
Schematic for a group of 256 correlators.

The correlation operation between two input signals essentially comprised multiplication and integration of the products of all samples. The architecture of the correlator is shown in Figure 10. The multiplication of two-level inputs was calculated simply by an exclusive NOR (XNOR) operation. Because a 1:4 serial-to-parallel conversion was performed on each data line, four 1-bit products were generated at a divided clock cycle, which were summed together and then forwarded to the integrator. To guarantee enough integration time for imaging and system calibration, the output width of the integrator was 36 bits, where the integration time could be up to 68 seconds at 1 GS/s. Considering the higher output bits of an integrator transition at a slower frequency than the lower output bits, we adopted a two-stage architecture for the integrator to minimize the cost of fabric resource. In the first stage, only an accumulator with 15-bit output was implemented using fabric resource for each integrator; in the second stage, we used a DSP slice together with a 512 × 36 dual-port random access memory (DPRAM) in a multiplexed approach to integrate the results from 256 first stage integrators [28]. The DPRAM was used in a ping-pong configuration, with only 256 locations being used at a time, sparing the other 256 for computer read operation, which made it possible to read earlier data while simultaneously performing the next correlation. As a result, 256 correlators were grouped as a basic cell to build the correlation processing module, as shown in Figure 10, while 2304 correlators were actually deployed in the FPGA. The device utilization for 2304 correlators using KCU040 is shown in Table 1.

To make the most of the fabric resources, the counters used to calibrate the threshold offset of the comparators were achieved using the extra correlators with a different input configuration. The total number of one-bit samples could be obtained by doing self-correlation. The number of “ones” in one-bit samples could be obtained by doing a cross-correlation between the one-bit data and an artificial vector of “all ones”.

### 3.4. Power Detection

**Figure 11:**
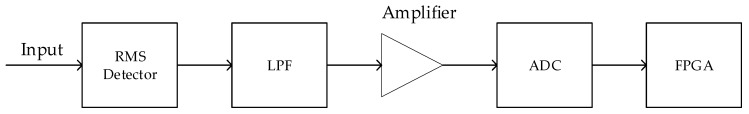
Schematic for power detection.

The schematic for power detection is illustrated in Figure 11, where a root mean square (RMS) detector was used to obtain the power information. The power detection result was sampled by an ADC and then led to an FPGA. Between the RMS detector and the ADC, a low-pass filter (LPF) was used to condition the signal bandwidth to fit the sampling frequency of the ADC, and an amplifier was used to amplify the signal to fit the dynamic range of the ADC. The digital power result could be integrated in the FPGA to obtain an integration time longer than that of the LPF, as shown in Figure 5.

To obtain a compact size, an integrated RMS detector LTC5581 [29] was used in our design, which outputs a DC voltage in linear scale proportional to an input signal power in dBm. For the large dynamic range of LTC5581, a 16-bit ADC LTC1864L [30] was used to guarantee quantization precision. The full-scale input voltage of LCT1864L was set to 1.2 V, and the input signal power of LTC5581 could be up to −5 dBm. The power measurement resolution was 6.41 × 10^−4^ dB per least significant bit (LSB), and the quantization error introduced by LTC1864L was less than $\frac{1}{2}$ LSB, which equals 0.045 Kelvin for a system noise temperature of 600 Kelvin. An LPF with 50 KHz bandwidth was added between LTC5581 and LTC1864L, for which the sampling frequency was 100 KHz.

## 4. Implementation and Test

**Figure 12:**
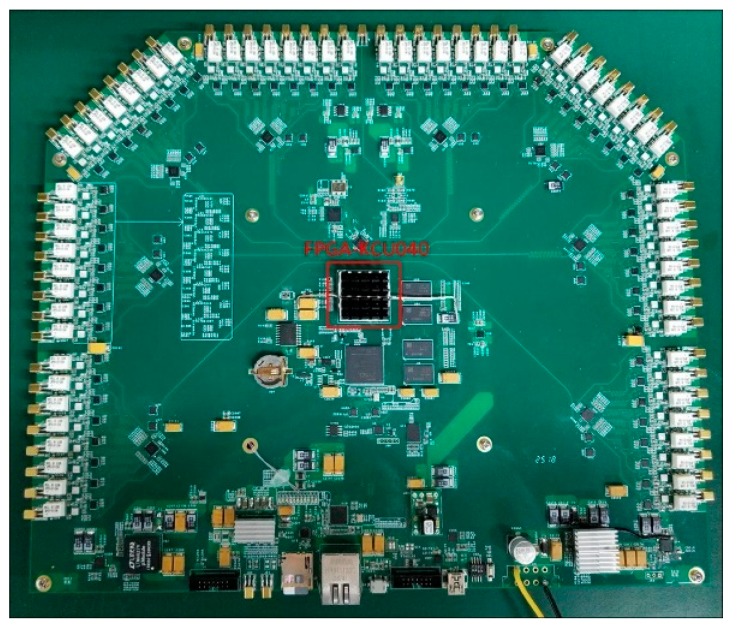
A 64-channel correlation module performing cross-correlation processing and power detection.

A photo of the 64-channel correlation board is shown in Figure 12, and the size is 305 × 265 mm^2^. The comparators and the power detectors were placed around the central FPGA. Along the edge of the PCB, MMCX connectors and power splitters were mounted. Since there were no other specific restrictions on this system, other components were selected accordingly. The entire power consumption was 48.75 W when the sampling rate was 1 GS/s.

### 4.1. Correlation Efficiency

**Figure 13:**
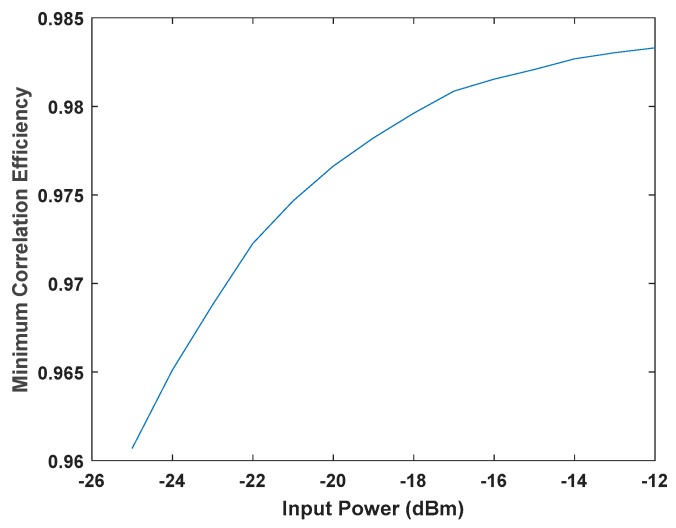
Minimum correlation efficiency measured at different input powers.

To characterize the effectiveness of the correlations, we measured the correlation efficiency as the ratio of the measured correlation coefficients to the ideal. This test was performed by feeding 100% correlated noise signals to all the input channels, where the ideal correlation coefficient was 1. Figure 13 shows the minimum correlation efficiency for different input powers, where the correlation efficiency exceeds 0.96 for input signals above −25 dBm.

### 4.2. Crosstalk

**Figure 14:**
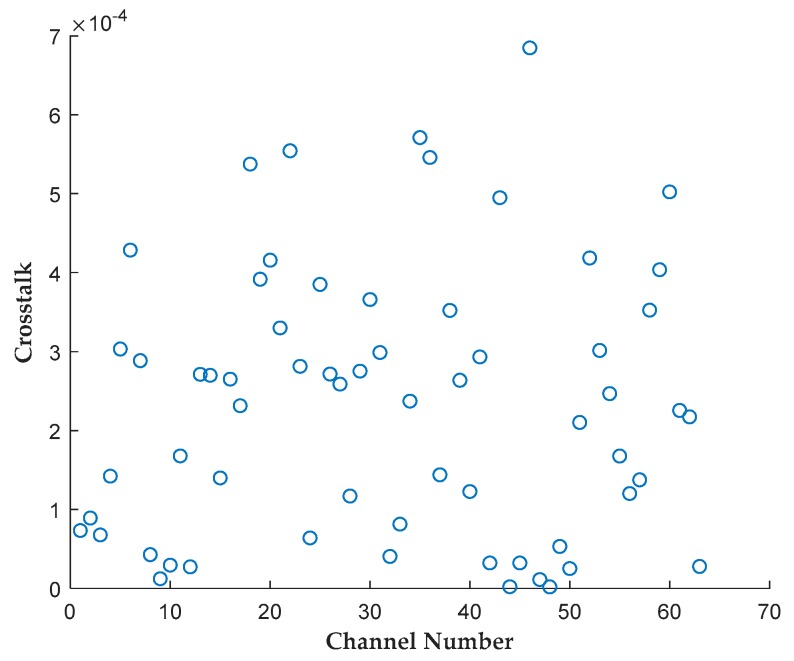
Crosstalk of every two adjacent channels.

Leakage from channels adjacent to the two input channels of a correlator cell causes the correlation result to have partial dependence on adjacent channels, which will greatly reduce the accuracy of measuring the small correlation coefficient. The crosstalk was measured by sending uncorrelated white noise signals to two neighboring channels and leaving all the other channels empty, where the correlation coefficient would ideally be zero. The absolute value of the correlation coefficients after calibrating the threshold offset of comparators was used as the measured crosstalk, which was less than 0.068%, as shown in Figure 14.

### 4.3. Stability

**Figure 15:**
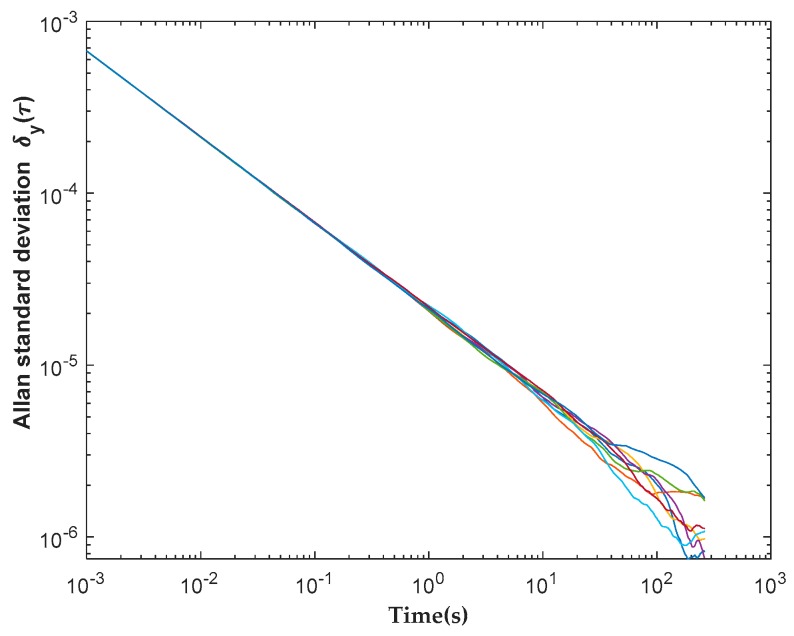
Allan standard deviation for eight cross-correlator channels.

The stability of the cross-correlator was assessed by measuring the Allen standard deviation [31] when the system views uncorrelated noise. As shown in Figure 15, a stability longer than 10 s was achieved. Because the longest integration time required for the imager is only 1 s in calibration mode, this cross-correlator system offers a large stability margin.

## 5. Conclusions

The design and implementation of a 1B/2L digital correlation system for interferometric aperture synthesis imaging was presented in this paper. The digitization was achieved using comparators, and the correlation processing was achieved using FPGA. Compared with the similar correlation system in [6], the sampling frequency was increased up to 1 GHz, which has great benefits for improving radiometric sensitivity. The methods for controlling the timing errors and threshold offsets of comparators were also presented, following which a high correlation efficiency can be obtained. Although this correlation system is designed for one-dimensional aperture synthesis, the design methodology has the potential to integrate many more channels on a larger FPGA, which is useful for 2-D aperture synthesis systems with a great number of channels.

## Figures and Tables

**Table 1:** Device Utilization for 2304 Correlators Implemented in XCKU040 Device.

Resource	Available	Utilization	Utilization %
CLB LUT	242400	71717	29.59
CLB Register	484800	133884	27.62
CARRY8	30300	4042	13.34
F7 MUX	121200	4232	3.49
F8 MUX	60600	195	0.32
Block RAM	600	8	1.33
DSP	1920	8	0.42
